# The chronic rhinosinusitis with nasal polyp patient journey in the United States and Europe

**DOI:** 10.1186/s13223-024-00879-7

**Published:** 2024-02-26

**Authors:** Jeremiah Hwee, Lauren Lee, Mark Small, Steven G. Smith, Victoria S. Benson, Shiyuan Zhang

**Affiliations:** 1 Value Evidence and Outcomes, GSK, Mississauga, ON Canada; 2 Adelphi Real World, Bollington, Cheshire, UK; 3 Clinical Sciences, GSK, Durham, NC USA; 4 Epidemiology, Value Evidence and Outcomes, GSK, Brentford, Middlesex UK; 5 Value Evidence and Outcomes, GSK, Collegeville, PA USA

**Keywords:** Chronic Rhinosinusitis, Nasal polyps, Quality of life, Smell

## Abstract

In this letter to the editor, we present questionnaire-based data assessing the patient journey of adults with moderate–severe Chronic Rhinosinusitis with Nasal Polyps (CRSwNP) in the USA and five European countries. These data highlight how long and difficult the patient journey with CRSwNP can be and how improved disease awareness among physicians could lead to more timely diagnosis and treatment, and hence improved management of patients.

## To the editor

The symptoms of chronic rhinosinusitis with nasal polyps (CRSwNP) are often poorly controlled owing to misdiagnosis or inappropriate treatment [1, 2], which can negatively impact health-related quality of life (HRQoL) [3]. As poor disease management may result from limited awareness of CRSwNP by primary care physicians (PCPs) [2, 4], better understanding of the CRSwNP patient journey may help PCPs to identify unmet medical needs in this patient population.

Using questionnaire-based data from the Adelphi CRSwNP Disease Specific Programme [5], this cross-sectional study assessed the journey of adults with moderate–severe CRSwNP in the USA [6] and five European countries (EUR5: France, Germany, Italy, Spain, UK) [7]. The survey included patient self-completed records and physician-completed records: patients with a physician-confirmed diagnosis of moderate–severe CRSwNP, who consulted specialists and PCPs (UK only), were surveyed once in 2018–2019; physicians provided information on patients as a consecutive sample (next five patients with moderate–severe CRSwNP). Physicians and patients both reported on the CRSwNP patient journey, including medical history, CRSwNP symptoms and diagnosis (matched sample); physicians also reported on current disease management [6, 7]. The survey was approved by the Western Institutional Review Board (WIRB; now WIRB-Copernicus Group IRB), Washington, USA (Study numbers: 1,187,074 [USA]; #1-1162676-1 [EUR5]).

Fifty-two USA physicians and 155 European physicians collected data from 251 to 820 patients, respectively. Demographic data are summarised in Table 1.

**Table 1:** Patient demographics and clinical characteristics according to the physician (consecutive sample)*

	USA (*n* = 251)	EUR5 (*n* = 820)
Age, years, mean (SD)	45.1 (16.8)	44.8 (14.7)
Sex, n (%)		
Male	147 (58.6)	474 (57.8)
Female	104 (41.4)	346 (42.2)
Smoking status, n (%)		
Non-smoker	183 (72.9)	417 (50.9)
Current smoker	15 (6.0)	142 (17.3)
Former smoker	42 (16.7)	199 (24.3)
Unknown	11 (4.4)	62 (7.6)
Most common comorbidities, n (%)		
Allergic rhinitis	165 (65.7)	349 (42.6)
Asthma	124 (49.4)	314 (38.3)
Hypertension	54 (21.5)	139 (17.0)
Charlson comorbidity index, mean (SD)	0.12 (0.4)	0.19 (0.7)
Number of visits for CRSwNP in last 12 months, mean (SD)	3.7 (2.8)	4.0 (3.1)
Nasal polyp score before treatment (scale 0–8), mean (SD)	*n* = 198 4.9 (2.2)	*n* = 742 4.7 (1.9)
Physician-perceived severity before initiation of treatment for nasal polyps, n (%)		
Mild	6 (2.4)	28 (3.4)
Moderate	95 (37.8)	423 (51.6)
Severe	111 (44.2)	345 (42.1)
Don’t know	39 (15.5)	24 (2.9)

*Patient demographics and clinical characteristics were captured using a physician-reported patient report form

CRSwNP, chronic rhinosinusitis with nasal polyps; EUR5, five European countries (France, Germany, Italy, Spain, UK); SD, standard deviation

**Fig. 1:**
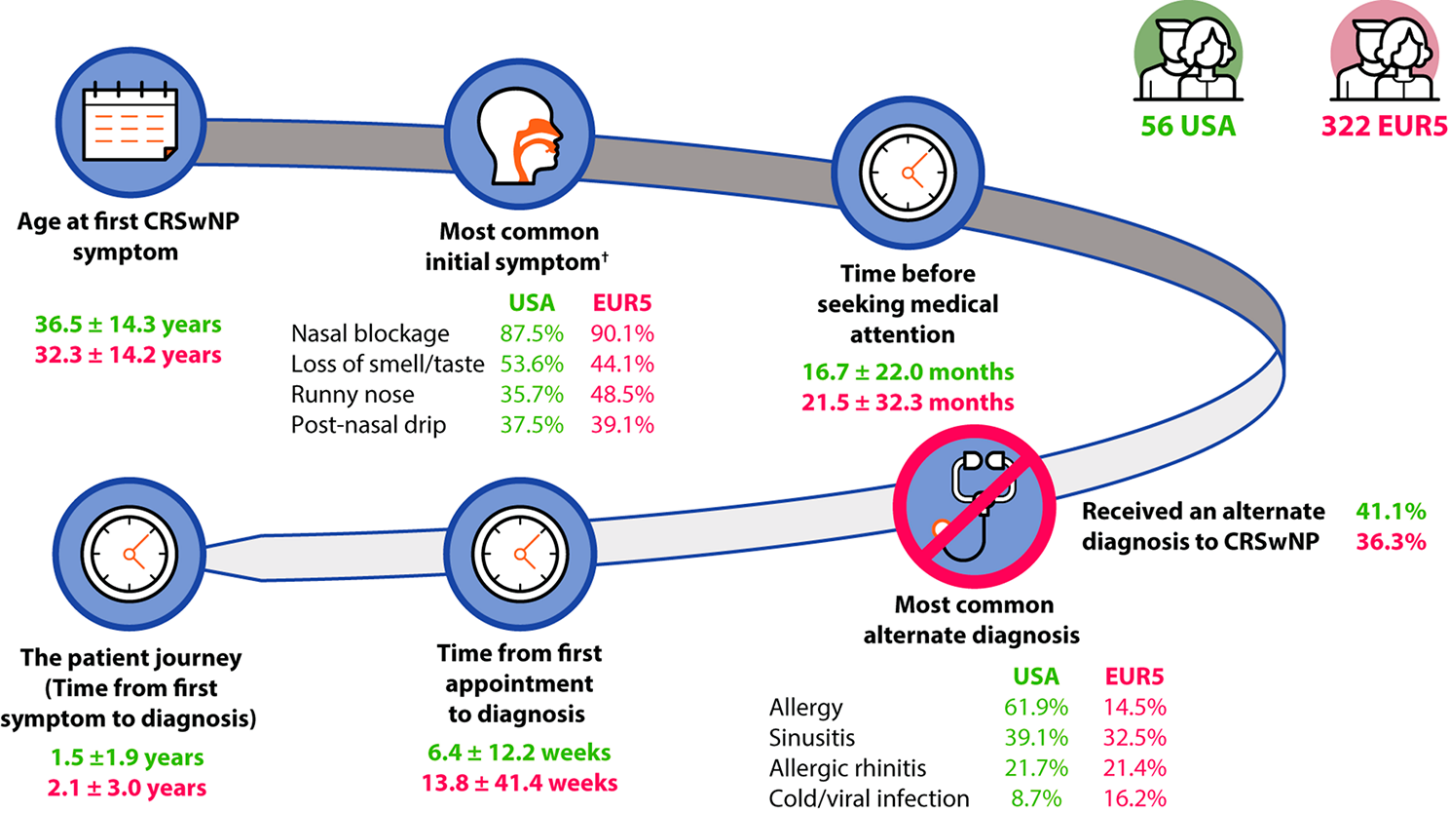
The CRSwNP patient journey according to patients (matched sample)*. *Data regarding the patient journey from the patient’s perspective were captured using a voluntary patient self-completed questionnaire. ^†^Prior to confirmed CRSwNP diagnosis. As this questionnaire was voluntary, questionnaires were not completed by every patient; 56 patients in the USA population and 322 patients in the EUR5 population answered every question on the questionnaire. The mean values are indicated for all time data. CRSwNP, chronic rhinosinusitis with nasal polyps; EUR5, five European countries (France, Germany, Italy, Spain, UK)

Figure 1 describes the patient-reported journey in CRSwNP where both the patient and physician completed the questionnaire (378/1071, 35.3%). The most common symptoms before CRSwNP diagnosis were nasal blockage, loss of smell/taste, runny nose, and post-nasal drip in both regions. Patients in the USA versus EUR5 took less time to seek medical attention from first symptoms (mean [SD]: 16.7 [22.0] versus 21.5 [32.3] months). Some patients received an initial alternate diagnosis; the most common was allergy, sinusitis, and allergic rhinitis. The mean (SD) time from first CRSwNP symptoms to CRSwNP diagnosis was shorter in the USA (1.5 [1.9] years) versus EUR5 (2.1 [3.0] years).

**Fig. 2:**
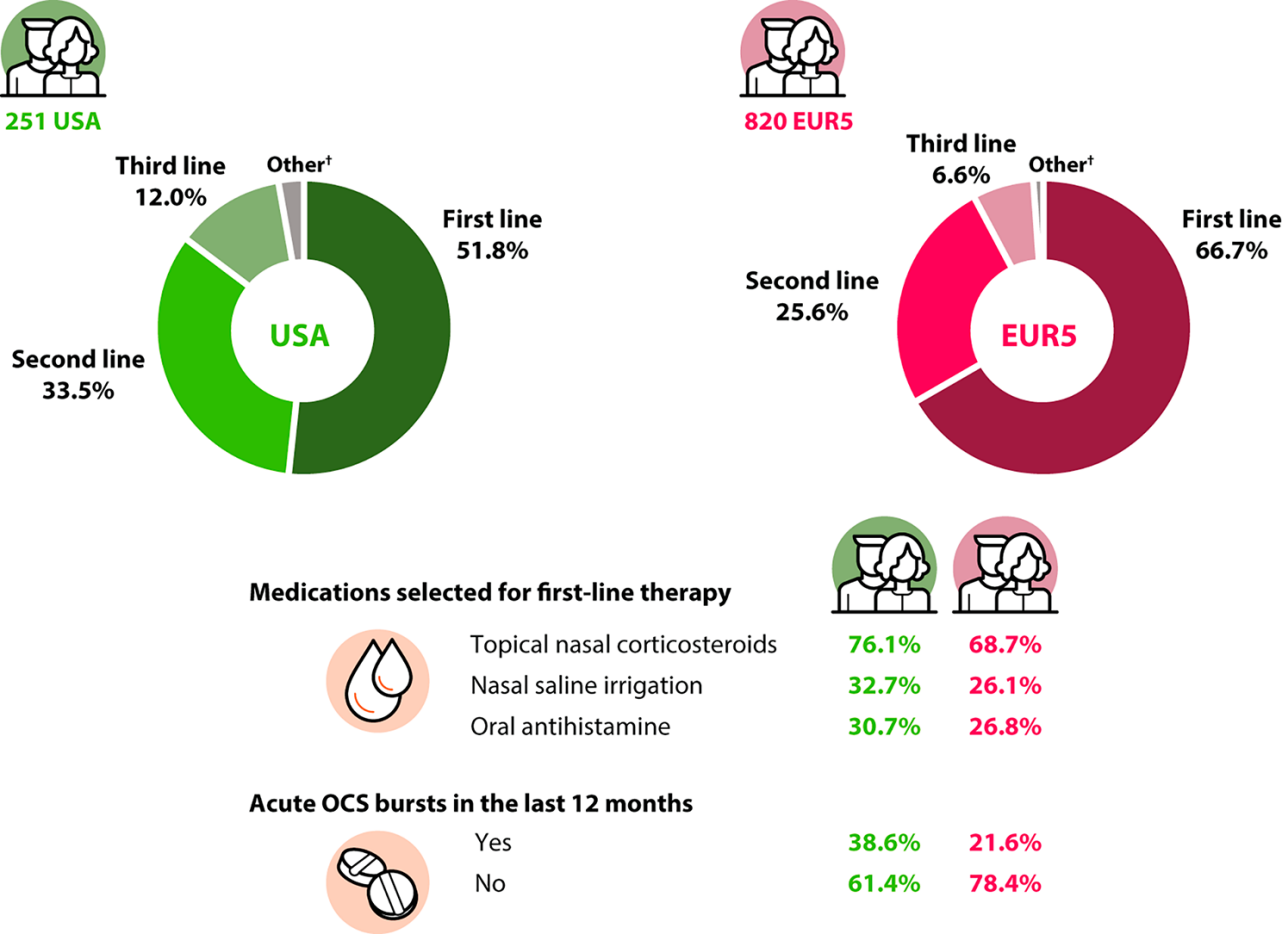
Current treatments for patients with CRSwNP according to the physician (consecutive sample)*. *Data regarding the patient journey from the physician’s perspective were captured using a physician-reported patient report form, which included current disease management. ^†^Other maintenance therapy included fourth-line (or higher) therapies. CRSwNP, chronic rhinosinusitis with nasal polyps; EUR5, five European countries (France, Germany, Italy, Spain, UK); OCS, oral corticosteroid

Physicians indicated 51.8% and 33.5% (USA) and 66.7% and 25.6% (EUR5) of all patients were receiving first- and second-line maintenance therapies, respectively; 38.6% (USA) and 21.6% (EUR5) received ≥ 1 oral corticosteroid (OCS) burst in the previous year for CRSwNP (Fig. 2), and 36.3% (USA) and 24.5% (EUR5) had ever undergone ≥ 1 sinus surgery.

Patients with CRSwNP endure a difficult and long patient journey, which can vary depending on geographical region. This may result from differences in healthcare systems and treatment approaches, or difficulties with arranging follow-up appointments with the same physician, leading to misdiagnosis, diagnosis delays, and inadequate treatment [8]. These findings are consistent with a Patient Advisory Board of European Forum for Research and Education in Allergy and Airway Diseases statement [4]. Patients with CRSwNP would like greater awareness from society and physicians on the disease burden of CRSwNP; patients were frustrated by the lack of coordinated care and the limited treatment options in CRSwNP [4]. Although treatments, such as OCS and sinus surgery, can improve HRQoL in CRSwNP, both lack long-lasting efficacy; nasal polyp recurrence following surgery is common for many patients (35–40%) [9], and OCS use is associated with adverse side effects [4].

Study limitations include an imbalanced sample size between regions, over-the-counter medicines not being captured, recall bias and potential selection bias, with patients with a moderate–severe phenotype more likely to consult physicians frequently than a general CRSwNP population. Although the patient selection process was not verified, selection bias was minimised using consecutive patient sampling. The EUR5 region consisted of 5 European countries, each with differing healthcare policies and practices. Overall, improved disease awareness among physicians could facilitate accurate diagnosis, enabling timely and appropriate treatment. This may improve HRQoL and reduce morbidity in CRSwNP.

## Data Availability

GSK makes available anonymized individual participant data and associated documents from interventional clinical studies that evaluate medicines, upon approval of proposals submitted to: https://www.gsk-studyregister.com/en/.

## Abbreviations

CRSwNP: chronic rhinosinusitis with nasal polyps
HRQoL: health-related quality of life
OCS: oral corticosteroid
PCP: primary care physician
SD: standard deviation
